# Rationale and design of the PHOspholamban RElated CArdiomyopathy intervention STudy (i-PHORECAST)

**DOI:** 10.1007/s12471-021-01584-5

**Published:** 2021-06-18

**Authors:** W. P. te Rijdt, E. T. Hoorntje, R. de Brouwer, A. Oomen, A. Amin, J. F. van der Heijden, J. C. Karper, B. D. Westenbrink, H. H. W. Silljé, A. S. J. M. te Riele, A. C. P. Wiesfeld, I. C. van Gelder, T. P. Willems, P. A. van der Zwaag, J. P. van Tintelen, J. H. Hillege, H. L. Tan, D. J. van Veldhuisen, F. W. Asselbergs, R. A. de Boer, A. A. M. Wilde, M. P. van den Berg

**Affiliations:** 1 Department of Genetics, University of Groningen, Groningen UMC, Groningen, The Netherlands; 2 Netherlands Heart Institute, Utrecht, The Netherlands; 3 Department of Cardiology, University of Groningen, Groningen UMC, Groningen, The Netherlands; 4 Department of Cardiology, Antonius Hospital, Sneek, The Netherlands; 5 Department of Clinical and Experimental Cardiology, Heart Center, Amsterdam UMC, University of Amsterdam, Amsterdam Cardiovascular Sciences, Amsterdam, The Netherlands; 6 Department of Cardiology, Division Heart & Lungs, Utrecht UMC, Utrecht, The Netherlands; 7 Department of Radiology, University of Groningen, Groningen UMC, Groningen, The Netherlands; 8 Department of Genetics, University of Utrecht, Utrecht UMC, Utrecht, The Netherlands; 9 Institute of Cardiovascular Science and Institute of Health Informatics, Faculty of Population Health Sciences, University College London, London, UK

**Keywords:** Cardiomyopathy, Intervention study, Phospholamban, Design, Pre-emptive treatment, Presymptomatic carriers

## Abstract

**Background:**

The p.Arg14del (c.40_42delAGA) phospholamban (PLN) pathogenic variant is a founder mutation that causes dilated cardiomyopathy (DCM) and arrhythmogenic cardiomyopathy (ACM). Carriers are at increased risk of malignant ventricular arrhythmias and heart failure, which has been ascribed to cardiac fibrosis. Importantly, cardiac fibrosis appears to be an early feature of the disease, occurring in many presymptomatic carriers before the onset of overt disease. As with most monogenic cardiomyopathies, no evidence-based treatment is available for presymptomatic carriers.

**Aims:**

The PHOspholamban RElated CArdiomyopathy intervention STudy (iPHORECAST) is designed to demonstrate that pre-emptive treatment of presymptomatic PLN p.Arg14del carriers using eplerenone, a mineralocorticoid receptor antagonist with established antifibrotic effects, can reduce disease progression and postpone the onset of overt disease.

**Methods:**

iPHORECAST has a multicentre, prospective, randomised, open-label, blinded endpoint (PROBE) design. Presymptomatic PLN p.Arg14del carriers are randomised to receive either 50 mg eplerenone once daily or no treatment. The primary endpoint of the study is a multiparametric assessment of disease progression including cardiac magnetic resonance parameters (left and right ventricular volumes, systolic function and fibrosis), electrocardiographic parameters (QRS voltage, ventricular ectopy), signs and/or symptoms related to DCM and ACM, and cardiovascular death. The follow-up duration is set at 3 years.

**Baseline results:**

A total of 84 presymptomatic PLN p.Arg14del carriers (*n* = 42 per group) were included. By design, at baseline, all participants were in New York Heart Association (NHYA) class I and had a left ventricular ejection fraction > 45% and < 2500 ventricular premature contractions during 24-hour Holter monitoring. There were no statistically significant differences between the two groups in any of the baseline characteristics. The study is currently well underway, with the last participants expected to finish in 2021.

**Conclusion:**

iPHORECAST is a multicentre, prospective randomised controlled trial designed to address whether pre-emptive treatment of PLN p.Arg14del carriers with eplerenone can prevent or delay the onset of cardiomyopathy. iPHORECAST has been registered in the clinicaltrials.gov-register (number: NCT01857856).

## Introduction

**Fig. 1:**
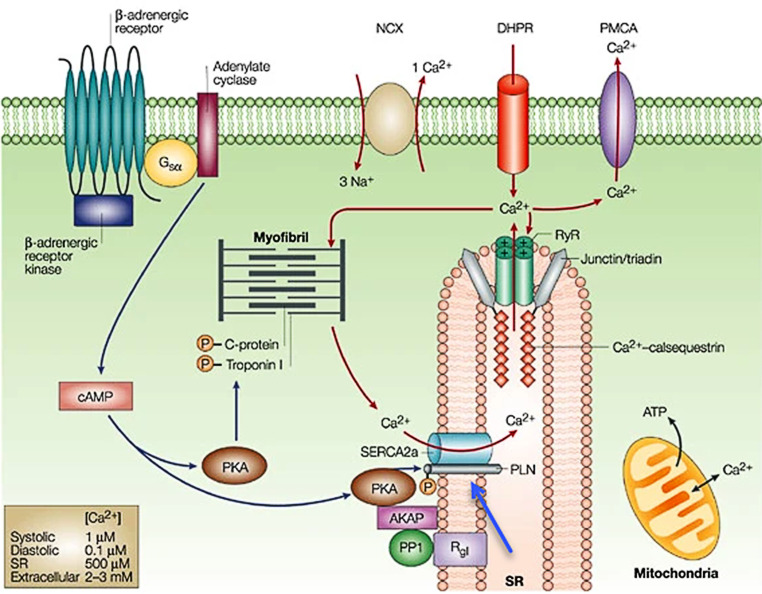
Function of phospholamban. Phospholamban (PLN; *blue arrow*) is a reversibly phosphorylated transmembrane protein that binds to and regulates the activity of the sarcoplasmic reticulum Ca^2+^ ATPase (SERCA2a) pump. *From*: MacLennan et al. Nat Rev Mol Cell Biol. 2003 [27]. Printed with permission from Springer Nature

In the Netherlands, approximately 15% of idiopathic dilated cardiomyopathy (DCM) patients and approximately 12% of arrhythmogenic right ventricular cardiomyopathy (ARVC) patients carry a single pathogenic variant, p.Arg14del (c.40_42delAGA), in the *PLN* gene that encodes the phospholamban protein, a regulator of cardiomyocyte calcium cycling (Fig. 1; [1]). The estimated prevalence of this founder mutation is approximately 1:500–1000 in large parts of the Netherlands. Moreover, it has been identified in several other European countries (Germany, Greece, Belgium, Norway, the UK and Spain), and in China, Canada and the United States of America, attesting to its global significance [2].

The clinical phenotype of PLN p.Arg14del carriers is characterised by malignant ventricular arrhythmias, sudden cardiac arrest and progressive heart failure [1, 3–5]. An experimental murine overexpression model of this PLN pathogenic variant found the cardiomyopathy to be characterised by progressive cardiomyocyte loss and severe cardiac fibrosis, and a similar phenotype was observed in a recently developed knock-in model that also recapitulated the clinical findings [6, 7]. Indeed, in our patients, we observed left ventricular myocardial fibrosis even in the presence of preserved (> 45%) left ventricular ejection fraction (LVEF), which suggests that tissue remodelling occurs early on in PLN p.Arg14del carriers, occurring even before onset of overt disease [1, 8, 9]. An earlier study by our group showed that an LVEF below 45% (rather than 35%, as in other types of cardiomyopathy) is an independent risk factor for ventricular arrhythmia in PLN p.Arg14del carriers [3]. We recently refined this observation, showing that left ventricular late gadolinium enhancement (LV-LGE) is an even stronger risk factor than LVEF. In fact, even when LVEF is preserved, LV-LGE appeared to be associated with a higher risk of ventricular arrhythmia in PLN p.Arg14del carriers [9].

Analogous to other inherited cardiomyopathies, the natural course of PLN p.Arg14del cardiomyopathy is age-related; after a presymptomatic phase of variable length, many PLN p.Arg14del carriers progress to overt cardiomyopathy. These patients are treated according to general clinical guidelines for these conditions.

Family cascade screening can, however, identify presymptomatic family members carrying the PLN p.Arg14del pathogenic variant, thereby providing a unique window of opportunity to initiate pre-emptive treatment in carriers before symptoms occur, potentially preventing sudden cardiac death or disease development and progression. However, no evidence-based treatment is currently available for presymptomatic carriers even though early treatment before the onset of any symptoms is potentially of major prognostic importance.

We reasoned that eplerenone, a selective mineralocorticoid receptor antagonist (MRA) with potent antifibrotic properties [10–16], might be effective as an early treatment for PLN p.Arg14del carriers. We are conducting the iPHORECAST trial to investigate whether treatment with eplerenone in presymptomatic PLN p.Arg14del carriers prevents or delays disease onset and symptoms.

## Study design

### Objectives

The objective of the iPHORECAST trial is to show that eplerenone treatment can prevent or reduce progression of disease in presymptomatic PLN p.Arg14del carriers.

### Study population

Specific inclusion and exclusion criteria are listed in Tab. 1. Previously, all known PLN p.Arg14del carriers in the Netherlands, including all presymptomatic carriers, were registered in the PHORECAST registry (PHOspholamban RElated CArdiomyopathy Study; http://www.phorecast.nl). Recently, all carriers have been incorporated in the national arrhythmogenic cardiomyopathy (ACM) registry (www.acmregistry.nl) [17]. We used the PHORECAST registry to recruit eligible presymptomatic subjects. Study participants are between 18 and 65 years of age, have a New York Heart Association (NYHA) functional class of 1 and an LVEF greater than 45% (as measured by cardiac magnetic resonance [CMR]). For ARVC and DCM criteria, we used the revised task force and Mestroni criteria respectively [18, 19]. Carriers taking other cardiovascular medication, including diuretics, angiotensin-converting enzyme (ACE) inhibitors, angiotensin receptor blockers, mineralocorticoid receptor antagonists, beta-blockers and anti-arrhythmic medication, were excluded.

**Table 1:** Key inclusion and exclusion criteria

*Key inclusion criteria*
PLN p.Arg14del carriers
Age ≥ 18 and ≤ 65 years
New York Heart Association functional class ≤ 1
LV ejection fraction ≥ 45 (measured with MRI)
*Key exclusion criteria*
Palpitations necessitating treatment (at the discretion of the attending physician)
A diagnosis of DCM (according to the Mestroni criteria [19]). Note: regional LV wall motion abnormalities are acceptable
A diagnosis of ARVC (according to the task force criteria [18])
Global or regional RV dysfunction and/or structural alterations (according to task force criterion 1 [18])
Ventricular premature complexes > 2500 during 24-hour Holter monitoring
Non-sustained ventricular tachycardia during Holter monitoring or exercise testing
History of sustained ventricular tachycardia or ventricular fibrillation
Hypertension requiring the use of antihypertensive drugs, or when this is anticipated within the coming 3 years
Evidence of ischaemic heart disease
Treatment with cardioactive medication
Hyperkalaemia (serum potassium > 5.0 mmol/l)
Severe renal dysfunction (eGFR < 30 ml/min/1.73 m^2^)
Severe hepatic impairment (Child-Pugh class C)
Women who are currently pregnant or report a recent pregnancy (last 60 days) or plan on becoming pregnant
Concomitant use of CYP3A4 inhibitors
Concomitant use of NSAIDs
Concomitant use of potassium-sparing agents
Known intolerance or contraindication for aldosterone antagonists
Participation in another drug trial in which the last dose of drug was within the past 30 days
Contra-indications for CMR (claustrophobia, metal devices)
Subjects unable or unwilling to provide written informed consent

Note: presence of late gadolinium enhancement on CMR is not an exclusion criterion

*PLN* phospholamban*, DCM* dilated cardiomyopathy,* LV* left ventricular, *ARVC* arrhythmogenic right ventricular cardiomyopathy,* RV* right ventricular,* eGFR* estimated glomerular filtration rate,* NSAIDs* non-steroidal anti-inflammatory drug*s, CMR* cardiac magnetic resonance

### Design

This outpatient study is a multicentre, prospective, randomised trial with blinded assessment of endpoints design (PROBE) [20]. The participating centres are the University Medical Center Groningen (which is the coordinating centre and CMR core lab); Amsterdam University Medical Center; Antonius Hospital Sneek and University Medical Center Utrecht. Carriers were randomly assigned to one of the two study arms, either eplerenone or control (no treatment), in a 1:1 ratio. The follow-up period is 3 years (see the section *Study endpoints*).

### Ethical considerations

This study is conducted in accordance with the principles stated in the revised Declaration of Helsinki (Seoul 2008) and in accordance with the Medical Research Involving Human Subjects Act. The medical ethics committee of the University Medical Center Groningen, the Netherlands, approved this study (METc 2013-272; ABR NL43771.042.13; EudraCT 2013-001067-23). The study protocol was also submitted to and approved by the medical ethics committee of each participating hospital. Written informed consent was obtained from every subject before entry into the study. An independent data and safety monitoring board (DSMB) safeguards the interests of i‑PHORECAST trial participants, assesses the safety of the interventions during the trial and monitors the overall conduct of the clinical trial. The study has been registered and kept up-to-date in the clinicaltrials.gov-register (number: NCT01857856).

### Treatment

In the treatment arm, eplerenone was initiated on the day of randomisation at 50 mg orally once daily. The dose of 50 mg once daily is the recommended dose in patients with heart failure. Carriers are maintained at this target dose, or the maximum tolerated dose, until the end of the study. Serum potassium and estimated glomerular filtration rate (eGRF) are monitored at the start of the study, 1–2 week(s) after study start and yearly, and these measures are documented together with the other investigations and vital parameters. The dose is reduced if needed, i.e. in case of hyperkalaemia (serum potassium > 5.5 mmol/l). Carriers undergo yearly follow-up in the cardiology outpatient clinic at each participating centre following routine clinical care. Physical examination, blood sampling (when treated with eplerenone), electrocardiogram, Holter monitoring and ergometry are performed every 12 months. CMR imaging and SA-ECG are performed at the start and end (36 months) of the study (Tab. 2).

**Table 2:** Overview of study procedures

	Visit 1 start	Visit 2 1 week	Visit 3 1 year	Visit 4 2 years	Visit 5 3 years
Visit to outpatient clinic	*✔*	**–**	*✔*	*✔*	*✔*
History and current status	*✔*	**–**	*✔*	*✔*	*✔*
Physical examination	*✔*	**–**	*✔*	*✔*	*✔*
Electrocardiogram	*✔*	–	*✔*	*✔*	*✔*
Echocardiogram	*✔*	–	*✔*	*✔*	*✔*
Holter monitoring	*✔*	–	*✔*	*✔*	*✔*
Ergometry	*✔*	–	*✔*	*✔*	*✔*
Signal-averaged ECG	*✔*	–	–	–	*✔*
CMR	*✔*	–	–	–	*✔*
Blood analysis	*✔*	*✔*^a^	*✔*^a^	*✔*^a^	*✔*

Note: Subjects undergo several investigations as summarised in this table. They are requested to visit the outpatient clinic at baseline and at 1‑year intervals thereafter. Subjects randomised to eplerenone visit the outpatient clinic or their general practitioner after 1 week to check serum potassium and eGRF, which are also checked in these subjects at the yearly visits. Of note, all investigations except blood analysis are already performed as part of routine clinical care, which routinely includes evaluation of signs and symptoms of DCM and ACM. For the purpose of the study, we collect and use these available data.

*ECG* electrocardiography, *CMR* cardiac magnetic resonance, *eGFR* estimated glomerular filtration rate, *DCM* dilated cardiomyopathy, *ACM* arrhythmogenic cardiomyopathy

^a^ only for subjects randomised to eplerenone

### Study endpoints

The primary and secondary endpoints are listed in Tab. 3. The primary endpoint is a multiparametric assessment of disease progression defined as a responder-based composite primary endpoint (Tab. 3). If one of the predefined parameters of the composite primary endpoint is reached, CMR imaging is performed and study end date recorded. After a follow-up period of 3 years, outcomes will be assessed by an independent adjudication committee blinded to the allocated group. In case of a favourable trend (but no significant effect as yet), the DSMB may advise continuing the study for 2 more years (“adaptive design”).

**Table 3:** Primary and secondary endpoints

*Primary endpoint*^a^
LV end-diastolic volume, increase > 10% (assessed with CMR)
LV ejection fraction, absolute decrease > 5% (assessed with CMR)
RV end-diastolic volume, increase > 10% (assessed with CMR)
RV ejection fraction, absolute decrease > 5% (assessed with CMR)
Late enhancement, absolute increase > 5% (assessed with CMR)
QRS voltage, decrease > 25% (ECG, measured in I, II and III in mV)
Occurrence of non-sustained ventricular tachycardia (Holter monitoring, exercise testing)
Heart failure signs and/or symptoms or arrhythmias necessitating medical treatment according to the guidelines and likely due to arrhythmogenic cardiomyopathy
*Secondary endpoints*
All individual components of the primary endpoint
Diagnosis of DCM (according to Mestroni criteria [19])
Diagnosis of ARVC (according to revised task force criteria [18])
Development of global or regional dysfunction and structural alterations (according to revised task force criterion 1 [18])
QRS axis (12-lead ECG)
Conduction intervals (PR interval, QRS duration [12-lead ECG; SA-ECG])
STT segment changes (12-lead ECG)
Change in biomarkers
Occurrence of sustained ventricular tachycardia or ventricular fibrillation
Hospitalisation for a cardiovascular reason

*LV* left ventricular, *CMR* cardiac magnetic resonance*, RV* right ventricular,* ECG* electrocardiogram,* DCM* dilated cardiomyopathy, *ARVC* arrhythmogenic right ventricular cardiomyopathy,* SA-ECG* signal-averaged electrocardiography

^a^The primary endpoint is a multiparametric assessment of disease progression defined as a responder-based composite primary endpoint. If one of the predefined parameters of the composite primary endpoint is reached, CMR imaging is performed and study end date recorded

### Sample size estimation

This clinical trial was designed based on the assumptions that 50% of the presymptomatic PLN p.Arg14del carrier group without treatment will reach the primary endpoint within 3 years and that treatment with eplerenone is expected to reduce this percentage by 50%. To ensure at least 80% power to detect a difference between the control group and the eplerenone group, at a significance level of 0.05, 128 carriers (64 in each group) are needed. To account for 10–15% loss to follow-up during the study, we aimed to include 150 subjects.

### Randomisation and blinding

Central randomisation was conducted by the Trial Coordination Center of the University Medical Center Groningen (www.tcc.umcg.nl). Randomisation was stratified according to participating centres to ensure a similar sample distribution between the two test groups. According to the PROBE design, randomisation is blinded to the CMR core lab in the UMCG for assessment of the CMR images, which are performed as previously described [9]. An independent endpoint adjudication committee assesses the other primary and secondary endpoints.

### Study organisation and monitoring

This is an investigator-initiated study organised by the executive steering committee, which is based in the Department of Cardiology, University Medical Center Groningen, Groningen, the Netherlands. The executive steering committee is responsible for overall supervision of the study, policy decisions, protocol amendments, publications and presentations. An independent DSMB monitors the overall conduct of this study by receiving and reviewing reports of serious adverse events, reviewing periodic reports of safety data and establishing ‘stopping rules’ for the trial for safety reasons only.

### Biomarkers

Venous blood samples from all participants are stored and analysed for levels of biomarkers of fibrosis [21] and for biomarkers of apoptosis, haemodynamic status and inflammation. We will investigate whether these biomarkers predict disease progression. In addition to the volume of blood needed to measure these biomarkers, additional blood (serum and plasma) is taken and stored to allow for additional analyses in the future (samples stored for a maximum of 15 years). The amount of blood taken per venipuncture is 20 ml (at the start of the study and after 3 years). Carriers were asked to give their written informed consent for these additional analyses at the start of the study.

### Statistical analysis

After completion of the study, all eligible carriers who have been randomised to the treatment arm and received at least 1 dose of eplerenone will be used in the analyses of the primary and secondary efficacy endpoints, as well as in the safety analysis. Safety will be assessed by summarising the incidence and type of adverse events and the changes in laboratory parameters. The proportion of carriers experiencing serious adverse events and the proportion of carriers with noteworthy changes in laboratory parameters will be compared between treatment groups. The analysis of primary endpoints and secondary endpoints will be performed according to their assigned treatment group in accordance with the intention-to-treat principle. Additional analysis sets may be used in exploratory analyses. The number of subjects withdrawing from the study will be tabulated by their reasons for withdrawal and by treatment group. Potenzial biases due to withdrawal of subjects will be investigated. Baseline characteristics will be compared using chi-squared test (or Fisher’s exact test if appropriate). If differences are found, caution will be exercised when interpreting the results of the analyses between groups and methods may be modified to adjust for this difference.

The primary outcome will be presented by Kaplan-Meier curves for the treatment group, followed by the stratified log-rank test using stratification variables. Multivariate analysis will also be used to compare the endpoints between the two groups, adjusting for other variables including sex, age and screening centre. For missing data, the last observations made will be used. Unless stated otherwise, all secondary analyses will be performed using the same subjects included in the primary analysis. Secondary variables will be analysed using an appropriate statistical test, depending on the nature of the variable. Changes in parameters over time in the different treatment groups will be analysed using repeated measurement analysis or techniques that evaluate the timing of endpoints, when appropriate. For all tests, a *p*-value < 0.05 is considered statistically significant. Statistical analysis will be performed in SPSS (IBM SPSS Inc., Chicago, IL, USA; newest version at moment of analysis).

## Baseline results

In May 2014, the first participant was included into the study. Because of slow recruitment, in January 2018 it was decided to stop further inclusion of participants and complete the protocolled follow-up duration with the included cohort (*N* = 84). At this point, it had become clear that it was not realistic to assume that enough participants would be included to reach the originally defined composite primary endpoint (Tab. 3). Instead of a composite dichotomous endpoint, i.e. using the pre-specified cut-off values, individual components of the primary endpoint will now be analysed on a continuous scale to investigate possible significant differences. The statistical analysis plan was amended accordingly, and the decision to amend the study design, i.e. to stop inclusion of participants and to continue with the included cohort, was supported by the DSMB and the medical ethics committee. The primary objective remains showing that eplerenone retards disease progression. The majority of participants have now finished the study and the remaining participants are expected to finish in 2021.

**Fig. 2:**
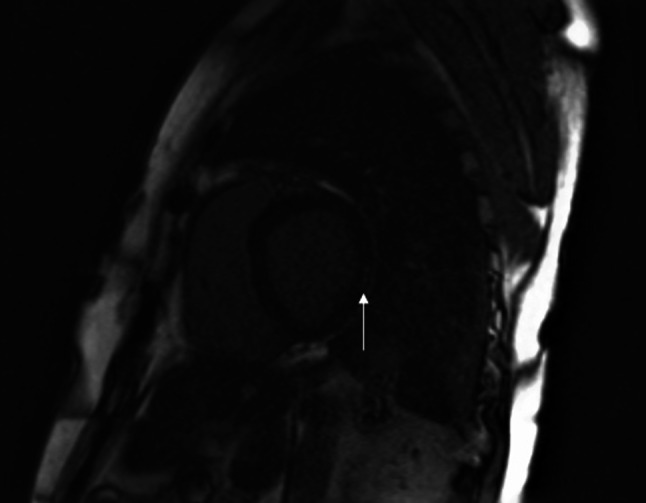
Left ventricular delayed contrast enhancement in PLN p.Arg14del carrier. Example of left lateral delayed contrast enhancement (*arrow*; short axis CMR image) in the left ventricle of PLN p.Arg14del carrier. *CMR* cardiac magnetic resonance, *PLN* phospholamban

An overview of the baseline characteristics of the 84 participants is provided in Tab. 4. By design, at baseline, all participants were in NHYA class I and had an LVEF > 45% and less than 2500 ventricular premature complexes (VPCs) during 24-hour Holter monitoring. Mean LVEF was 60% (±0.04). The median number of VPCs was 8 (0-1861). Baseline CMR scans were obtained for all 84 participants, but image quality was insufficient to perform adequate analyses in six cases. In the remaining 78 cases, as many as 14 cases (18%) showed LGE, mainly in the inferolateral left ventricular wall, suggestive of myocardial fibrosis. The six cases with insufficient image quality will be included for analysis of the other endpoints. An illustrative example is shown in Fig. 2. There were no statistically significant differences between the two groups in any of the baseline characteristics.

**Table 4:** Baseline characteristics: no statistical differences between eplerenone and control group

	All	Eplerenone group	Control group
Number, *n*	84	42	42
Age at inclusion, years (SD)	39.5 (±13.1)	40.2 (±13.0)	38.8 (±13.4)
Male sex, *n* (%)	37 (44)	18 (43)	19 (45)
Body mass index, kg/m^2^ (SD)	25.7 (±4.8)	26.5 (±5.6)	24.9 (±3.6)
Systolic blood pressure, mm Hg (SD)	124 (±16)	128 (±17)	121 (±13)
Diastolic blood pressure, mm Hg (SD)	74 (±11)	75 (±10)	74 (±11)
ECG repolarisation abnormalities, *n* (%)	18 (21)	13 (31)	5 (12)
VPC, *n* (range)	8 (0–1861)	8 (0–1498)	12 (0–1861)
LVEDV, ml (SD)	157 (±28)	160 (±23)	154 (±34)
LVEF, % (SD)	60 (±0.04)	60 (±0.04)	59 (±0.05)
LV-LGE, *n* (%)	14 (18)	9 (22)	5 (13)
RVEDV, ml (SD)	178 (±34)	179 (±27)	176 (±42)
RVEF, % (SD)	52 (±0.04)	52 (±0.04)	51 (±0.04)

*SD* standard deviation*, ECG* electrocardiogram, *VPC* ventricular premature complexes, *LVEDV* left ventricular end-diastolic volume, *LVEF* left ventricular ejection fraction, *LV-LGE* left ventricular late gadolinium enhancement, *RVEDV* right ventricular end-diastolic volume, *RVEF* right ventricular ejection fraction

## Discussion

The PLN p.Arg14del founder mutation causes DCM and ACM and is associated with an increased risk of malignant ventricular arrhythmia and heart failure [1, 8, 9]. Although DCM and ACM are considered separate entities by both the American Heart Association [22] and the European Society of Cardiology [23], they do have overlapping clinical features [24–26]. This is particularly true in PLN p.Arg14del cardiomyopathy, where we find significant clinical overlap [1].

The multicentre prospective randomised clinical iPHORECAST study aims to show that preventive treatment (eplerenone) can prevent or delay disease onset in presymptomatic PLN p.Arg14del carriers. Given eplerenone’s potent antifibrotic effects, we anticipate it may retard disease progression. Eplerenone has been shown to be well tolerated with minimal side-effects, even in advanced heart failure patients. The most significant side-effect is hyperkalaemia, which mainly occurs in combination with other potassium-sparing drugs and/or renal impairment. The presymptomatic subjects in the present study, who all have normal kidney function are at very low risk for these side-effects. By using strict inclusion and exclusion criteria and follow-up monitoring, we are convinced that the use of eplerenone in the present study is safe.

The primary objective of this study is to assess the effect of eplerenone on disease onset and progression. To comply with daily clinical practice and demonstrate overall disease progression, a composite endpoint of CMR parameters (CMR volumes, systolic function and fibrosis), electrocardiographic parameters (QRS voltage, ventricular ectopy), signs and/or symptoms related to DCM and ACM, and cardiovascular death is adopted as the primary endpoint. Serum markers of fibrosis, apoptosis, haemodynamic status and inflammation are measured to investigate whether they can predict disease progression. Of note, as discussed in the baseline results, the actual number of participants was lower than projected. As a result, we will now perform an adapted assessment of the primary endpoint but are hopeful that meaningful results will be obtained. As to their clinical characteristics, the participants are a good reflection of clinical practice; indeed, many of these presymptomatic PLN p.Arg14del carriers already have subtle signs of disease, including repolarisation abnormalities of the ECG and frequent VPCs. Moreover, LGE on CMR, indicative of fibrosis, was observed in as many as 18% of the participants at the start of the study thereby supporting the rationale to study the effect of eplerenone.

In conclusion, iPHORECAST is a multicentre, randomised, controlled trial, designed to examine whether treatment with eplerenone in presymptomatic PLN p.Arg14del carriers can prevent or delay disease onset.
